# Anxiety and Motivation to Return to Sport During the French COVID-19 Lockdown

**DOI:** 10.3389/fpsyg.2020.610882

**Published:** 2020-12-17

**Authors:** Alexis Ruffault, Marjorie Bernier, Jean Fournier, Nicolas Hauw

**Affiliations:** ^1^Laboratory “Sport, Expertise, and Performance” (EA 7370), French Institute of Sport (INSEP), Paris, France; ^2^Unité de Recherche Interfacultaire Santé et Société, Université de Liège, Liège, Belgium; ^3^CREAD (EA 3875), Université de Brest, Brest, France; ^4^Laboratoire Interdisciplinaire en Neurosciences, Physiologie et Psychologie: Activité Physique, Santé et Apprentissages, Université Paris Nanterre, Nanterre, France; ^5^Institute of Physical Education and Sports Sciences (IFEPSA), West Catholic University of Angers, Angers, France

**Keywords:** anxiety, motivation, lockdown, return to sport, sport injury

## Abstract

Feeling anxious and presenting self-determined motivations about returning to sport after a break may impair sport performance and increase the risk of sustaining an injury. Hence, the aim of this study is to explore differences in anxiety and motivation to return to sport according to gender, expertise, training status before and during the lockdown, and athletes’ availability (i.e., injury status) at the time of the lockdown. A total of 759 competitive athletes (49% female; mean age: 27 ± 10 years old) completed the cross-sectional study. Participants were invited to state their expertise, training status before and during the lockdown (did they have a training program), and whether they were injured at the start of the lockdown. Additionally, participants filled out psychometric self-report measures of anxiety (TFAI-return) and motivation (SMS-return) to return to sport. Due to non-normal distributions in the TFAI and SMS scores, non-parametric group comparisons were performed to compare participants for each categorical variable: non-parametric correlation tests were also performed to test the associations between continuous variables. Group comparisons showed higher scores of anxiety for females, younger athletes, athletes practicing and competing at the highest level, and athletes without a training program during the lockdown. Moreover, results suggested lower motivation scores (i.e., autonomous and controlled) for older athletes, experts (practicing for more than 10 years), athletes practicing and competing at a lower level, and athletes without a training program during the lockdown. Additionally, participants who were injured at the start of the lockdown reported higher scores of cognitive anxiety to return to sport than non-injured participants. The results of this study suggest that elite athletes may have suffered from external pressures to return to sport during the lockdown. Additionally, participants with a training program during the lockdown seemed to be less anxious and more self-determined to return to sport after the lockdown. Future studies may focus on the impact of cognitive behavioral interventions on anxiety and motivation to return to sport.

## Introduction

In France, in reaction to the COVID-19 crisis, the government decided to lock the population down for more than 2 months starting in mid-March 2020. However, quarantine increases the risk of psychological distress (Brooks et al., 2020). A systematic review of the literature shows that the COVID-19 pandemic increased the prevalence of anxiety and mood disorders in the general population—33 and 28%, respectively (Luo et al., 2020). Additionally, quarantine prevents athletes from training in their habitual settings, and most national and international competitions were canceled or postponed until further notice. Hence, the lockdown created uncertainty and stress related to sport practice and future return to competitions may have increased athletes’ anxiety (Grupe and Nitschke, 2013) and impaired motivation to return to sport (McGregor et al., 2010). On the other hand, Lades et al. (2020) showed that daily activity (e.g., exercising) during the COVID-19 lockdown increased positive affects and decreased negative affects in 604 individuals from the general population in Ireland. Additionally, in Italy, Chirico et al. (2020) showed that higher anxiety scores during the COVID-19 lockdown negatively influenced intentions to adopt physical activity behaviors. This protective role of exercise as demonstrated in the general population may have also applied to athletes who maintained sport practice or physical training during the lockdown.

Anxiety and motivation are two psychological variables that are associated with the risk of injury and lower performance when returning to sport (Wiese-Bjornstal, 2019). The literature on return to sport following an injury posits that athletes may be anxious because they think they will not meet the level of their pre-injury performances, or because they may not meet their coaches’ expectations, among other reasons (Podlog and Eklund, 2007; Podlog et al., 2011). Moreover, motivational states from Self-Determination Theory (SDT; Ryan and Deci, 2017) are also psychological determinants of return to sport. A study of Podlog and Eklund (2010) with professional Australian Football players revealed that greater self-determination in the return to sport results in more positive appraisals and affect. Even if the literature on return to sport lies in the context of an injury and rehabilitation, the context of lockdown displays similar aspects: athletes could not train as they used to and were not competing. In fact, long periods of rest and the absence of competitions represent a significant risk for athletes’ health and performances (Paoli and Musumeci, 2020). Hence, examining the impact of a confinement period on self-determination and anxiety could be of great interest.

A study on perceived stress in Italian athletes showed that male athletes and elite athletes (i.e., competing at the national and/or international level) displayed lower perceived stress during the COVID-19 lockdown than female and non-elite athletes, respectively (di Fronso et al., 2020). Additionally, Clemente-Suárez et al. (2020) found lower rates of anxiety in Olympic and Paralympic athletes than in the general population, suggesting that athletes may have more cognitive and emotional resources to face the confinement situation (Costa et al., 2020). Furthermore, in a qualitative study investigating psychological correlates of return to competition in basketball players, participants stated that participation in training programs prior to returning to sport increased their confidence in having better performance and lower fear of reinjury, and that reaching their pre-injury levels of performance was the main motivation to give “100% effort” in training programs before returning to competition (Conti et al., 2019). These results show the importance of considering both anxiety and motivation when studying the psychological aspects of return to sport after a break.

The literature on psychological states of athletes during the COVID-19 crisis focused on perceived stress, anxiety, and coping resources, but not on motivation to return to sport. It is also not clear whether the level of competition or the training habits (before and during lockdown) differentiate between athletes’ psychological predictors of return to sport. Hence, the aim of this study is to explore differences in anxiety and self-determined motivation to return to sport according to gender, expertise, training status before and during the lockdown, and athletes’ availability (i.e., injury status) at the time of the lockdown.

## Materials and Methods

This study was performed in April and May 2020 using an online software for conducting surveys (Limesurvey GmbH, 2003). A correlational design with convenience sampling was used, advertising the survey on social media (Facebook, Twitter, and LinkedIn). During lockdown, this methodology was deemed appropriate for obtaining information in order to respect social distancing rules.

### Participants

In the present study, a total of 1302 individuals read the online informed consent disclosure, from which 1206 were screened for inclusion. Eligibility criteria were as follows: practicing sport in competition (1147 “yes”), being 18 years old or over (1123 “yes”), not being under the protection of social services (1144 “no”), and providing informed consent to participate (972 “yes”). Exclusion criterion was participants with missing data. A total of 759 completed the full survey (49% female; mean age: 27 ± 10 years old). More than 40 sports were represented: 270 participants practiced track and field, 43 triathlon, 38 basketball, 36 judo, 33 shooting, 31 soccer, 27 gymnastics, 24 handball, 24 cycling, 17 tennis, 17 skiing, 16 fencing, 16 swimming, 16 boxing, 15 badminton, 13 rowing, 12 table tennis, 11 ice-skating, 10 rugby, and 90 other sports.

Ethical review and approval was not required for the study on human participants in accordance with the local legislation and institutional requirements. Before participation, experimental procedures were explained online to all the participants who gave their voluntary online informed consent in accordance with the Declaration of Helsinki.

A total of 37 participants stated that they were injured at the start of the lockdown. The estimated duration of time loss after injury (*M* = 222 ± 196.3 days, including 169.9 ± 171.3 days before lockdown and 52.1 ± 58.0 days during lockdown) did not show any significant correlation with neither anxiety nor motivation to return to sport.

### Measurements

#### Personal and Sport-Related Information

In addition to their age and gender, participants were first asked a set of general information regarding their sport practice. We estimated participants’ expertise level by asking them if they practiced their sport for less than 10 years or 10 years or more. Participants were considered “elite athletes” if they present such an official national status (listed in the Sport Ministry repository) and “competitive athletes” if they practiced their sport in competition without the official elite status. Participants were asked for their number of training sessions per week (2 or less, 3 or 4, and more than 4) and their highest level of competition (regional, national, and international) before the lockdown. Additionally, the training status during the lockdown was estimated: participants had to state whether they had a training program during the lockdown or not.

Regarding injury, participants were asked whether they were injured at the start of the lockdown (not injured; injured). For injured participants, additional information was recorded: (1) estimated time loss duration (in days) and (2) adoption (no; yes) of a rehabilitation program during the lockdown.

#### Psychometric Questionnaires

Anxiety to return to sport was assessed using an adapted version of the Three-Factor Anxiety Inventory 2 (TFAI-2; Jones et al., 2019). The original version of the TFAI-2 is used to assess pre-competitive anxiety with three factors and six subscales: cognitive anxiety (worry, private self-focus, and public self-focus), physiological anxiety (somatic tension and autonomic hyperactivity), and perceived control. The general instructions were modified to assess anxiety to return to sport at the moment of study completion: “A sport break may have a strong impact. People can experience it very differently. Below is a list of athletes’ affirmations describing their psychological states when they return to sport after a break. This questionnaire measures how you feel at the moment.” Items measuring worry, private self-focus, and perceived control were modified to match concerns regarding “return to sport” instead of “performance.” Participants were asked to rate 25 items on a five-point Likert scale ranging from 1 (totally disagree) to 5 (totally agree).

Motivation to return to sport was assessed using an adapted version of the Sport Motivation Scale 2 (SMS-2; Pelletier et al., 2019). The original version of the SMS-2 assesses motivation to practice sport with six motivation subtypes from the SDT (intrinsic motivation, integrated, identified, introjected, and external regulation of extrinsic motivation, and amotivation). In the general instructions and in each item, elements such as “practicing sport” were replaced with “returning to sport” in order to contextualize motivations toward an upcoming “return to competition and/or training” instead of “sport participation.” Participants were asked to rate 18 items on a seven-point Likert scale ranging from 1 (do not agree) to 7 (completely agree).

Confirmatory factor analysis (CFA) was conducted using lavaan (Rosseel, 2012) in order to check whether the French versions of the TFAI-return and SMS-return were close to their original validated versions. The model fit of the TFAI-return and the SMS-return was assessed using the comparative fit index (CFI), the Tucker–Lewis index (TLI), and the root-mean-square error of approximation (RMSEA) as goodness-of-fit statistics. CFI and TLI values close to or above 0.90 and 0.95 were considered acceptable (Hu and Bentler, 1998); RMSEA values close to or below 0.08 were considered acceptable (Browne and Cudeck, 1992). The results of the CFA showed an acceptable fit of the factor structure of both psychometric questionnaires to our data. For the TFAI-return, fit indices were as follows: CFI = 0.84, TLI = 0.82, RMSEA = 0.07 (*p* < 0.001). For the SMS-return, fit indices were as follows: CFI = 0.88, TLI = 0.85, RMSEA = 0.08 (*p* < 0.001).

### Statistical Analysis

Based on the results of Shapiro–Wilk tests, non-parametric tests were used. To investigate between-group differences in anxiety and motivation, Wilcoxon tests with continuity correction were performed for categorical variables with two groups; Kruskal–Wallis tests were conducted for categorical variables with more than two groups. Kendall’s Tau correlation tests were used to explore the correlation between anxiety, motivation, and other continuous variables.

All analyses were performed using R (R: A Language and Environment for Statistical Computing, 2013). Participants with injured status during the time of the survey were analyzed separately.

## Results

Table 1 shows the number of participants (*N*) and percentage (%) for each categorical variable, and the mean (*M*) and standard deviation (*SD*) for continuous variables. Participants who were injured by the start of the lockdown are displayed separately from others.

**TABLE 1:** Descriptive statistics of the study variables.

	Not injured (*N* = 722)		Injured (*N* = 37)	
	*N* (%)	*M* (*SD*)	*N* (%)	*M* (*SD*)
**Age** (years)		27.23 (10.25)		24.08 (6.3)
**Gender:** Female	358 (49.6%)		16 (43.2%)	
**Gender:** Male	364 (50.4%)		21 (56.8%)	
**Expertise:** >10 years	431 (59.7%)		21 (56.8%)	
**Expertise:** <10 years	291 (40.3%)		16 (43.2%)	
**Elite:** Yes	128 (17.7%)		6 (16.2%)	
**Elite:** No	594 (82.3%)		31 (83.8%)	
**Weekly training:** >4	394 (54.6%)		21 (56.8%)	
**Weekly training:** 3 or 4	197 (27.3%)		11 (29.7%)	
**Weekly training:** 2 or less	131 (18.1%)		5 (13.5%)	
**Competition level:** International	127 (17.6%)		6 (16.2%)	
**Competition level:** National	255 (35.3%)		10 (27.0%)	
**Competition level:** Regional	340 (47.1%)		21 (56.8%)	
**Training program during lockdown:** yes	586 (81.2%)			
**Training program during lockdown:** no	136 (18.8%)			
**Rehabilitation program during lockdown:** yes			21 (56.8%)	
**Rehabilitation program during lockdown:** no			16 (43.2%)	

The comparison between injured (*N*_inj_ = 37) and non-injured (*N*_non–inj_ = 722) participants at the start of the lockdown suggested significantly higher scores of worry (*M*_inj_ = 3.64 ± 0.93; *M*_non–inj_ = 3.04 ± 0.98; *p* < 0.001), private self-focus (*M*_inj_ = 3.57 ± 0.68; *M*_non–inj_ = 3.28 ± 0.69; *p* < 0.05), cognitive anxiety (*M*_inj_ = 3.36 ± 0.64; *M*_non–inj_ = 3.04 ± 0.66; *p* < 0.01), introjected regulation of external motivation (*M*_inj_ = 5.45 ± 1.10; *M*_non–inj_ = 4.96 ± 1.21; *p* < 0.05), and intrinsic motivation (*M*_inj_ = 5.80 ± 1.13; *M*_non–inj_ = 5.45 ± 1.15; *p* < 0.05) for injured participants when compared to non-injured participants.

For non-injured participants, results showed significant negative correlations between age and: worry (τ = −0.14; *p* < 0.001), public self-focus (τ = −0.14; *p* < 0.001), cognitive anxiety (τ = −0.13; *p* < 0.001), external regulation of extrinsic motivation (τ = −0.07; *p* < 0.01), and identified regulation of extrinsic motivation (τ = −0.07; *p* < 0.01). Female participants reported significantly higher scores of cognitive and physiological anxiety, as well as significantly lower scores of perceived control than male participants (see Table 2). These results suggest that older participants had lower cognitive anxiety and extrinsic motivation to return to sport and that female participants were more anxious (both cognitively and physiologically) while perceiving less control over return to sport than male participants.

**TABLE 2:** Differences in anxiety and motivation between groups for gender and athlete official status.

	Gender			Athlete official status		
	Female (*N* = 358)	Male (*N* = 364)	*p*	Elite (*N* = 128)	Non-elite (*N* = 594)	*p*
Worry	3.25 ± 0.96	2.83 ± 0.96	<0.001	3.08 ± 1.01	3.03 ± 0.98	
Private self-focus	3.30 ± 0.70	3.27 ± 0.68		3.26 ± 0.70	3.29 ± 0.68	
Public self-focus	2.96 ± 0.94	2.67 ± 0.83	<0.001	3.08 ± 0.85	2.76 ± 0.90	<0.001
**Total cognitive anxiety**	3.17 ± 0.65	2.92 ± 0.65	<0.001	3.14 ± 0.63	3.02 ± 0.66	
Somatic tension	2.30 ± 0.84	2.15 ± 0.84	0.005	2.07 ± 0.72	2.26 ± 0.86	
Autonomic hyperactivity	1.89 ± 0.74	1.74 ± 0.72	0.003	1.75 ± 0.64	1.83 ± 0.75	
**Total physiological anxiety**	2.09 ± 0.73	1.94 ± 0.72	0.002	1.91 ± 0.62	2.04 ± 0.75	
**Perceived control**	4.04 ± 0.67	4.27 ± 0.58	<0.001	4.15 ± 0.71	4.16 ± 0.62	
Amotivation	1.54 ± 0.87	1.47 ± 0.79		1.58 ± 0.89	1.48 ± 0.82	
External regulation	3.09 ± 1.54	3.12 ± 1.51		3.55 ± 1.55	3.01 ± 1.50	<0.001
Introjected regulation	5.01 ± 1.15	4.92 ± 1.28		4.90 ± 1.23	4.98 ± 1.21	
Identified regulation	5.47 ± 1.09	5.47 ± 1.08		5.53 ± 1.01	5.45 ± 1.10	
Integrated regulation	5.83 ± 0.97	5.79 ± 0.96		5.90 ± 0.95	5.79 ± 0.97	
Intrinsic motivation	5.38 ± 1.20	5.51 ± 1.10		5.51 ± 1.13	5.43 ± 1.15	

*Values are represented as mean ± standard deviation.*

Elite participants from the Sport Ministry list showed significantly higher scores of public self-focus and external regulation of extrinsic motivation to return to sport than non-elite participants (see Table 2). Additionally, participants with a higher level of competition (international and national) reported significantly higher scores of cognitive anxiety (including public self-focus), as well as significantly higher scores of both autonomous and controlled motivations (excepted for amotivation) to return to sport than regional competitors (see Table 3). These results suggest that participants competing at the highest level of competition were mainly concerned by the perception of the public when they return to sport, and more motivated for external (e.g., rewards they will receive when returning to sport) and internal (e.g., desire to improve or master their sport) reasons, or pleasure.

**TABLE 3:** Differences in anxiety and motivation between groups for expertise and competition level.

	Expertise			Competition level			
	>10 years (*N* = 431)	<10 years (*N* = 291)	*p*	International (*N* = 127)	National (*N* = 255)	Regional (*N* = 340)	*p*
Worry	3.00 ± 1.00	3.10 ± 0.96		2.99 ± 0.96	3.14 ± 0.97	2.98 ± 1.00	
Private self-focus	3.24 ± 0.72	3.35 ± 0.62	0.034	3.18 ± 0.71	3.32 ± 0.67	3.29 ± 0.69	
Public self-focus	2.82 ± 0.91	2.80 ± 0.89		3.01 ± 0.87	2.91 ± 0.88	2.67 ± 0.90	<0.001
**Total cognitive anxiety**	3.02 ± 0.67	3.08 ± 0.64		3.06 ± 0.63	3.12 ± 0.65	2.98 ± 0.67	0.027
Somatic tension	2.21 ± 0.83	2.25 ± 0.86		2.18 ± 0.82	2.27 ± 0.85	2.20 ± 0.84	
Autonomic hyperactivity	1.81 ± 0.73	1.83 ± 0.74		1.78 ± 0.63	1.88 ± 0.77	1.78 ± 0.74	
**Total physiological anxiety**	2.01 ± 0.72	2.04 ± 0.74		1.98 ± 0.67	2.08 ± 0.74	1.99 ± 0.74	
**Perceived control**	4.15 ± 0.63	4.16 ± 0.65		4.21 ± 0.65	4.17 ± 0.63	4.12 ± 0.63	
Amotivation	1.52 ± 0.85	1.48 ± 0.80		1.58 ± 0.88	1.52 ± 0.88	1.46 ± 0.77	
External regulation	3.09 ± 1.55	3.13 ± 1.49		3.43 ± 1.58	3.26 ± 1.51	2.87 ± 1.47	<0.001
Introjected regulation	4.97 ± 1.20	4.95 ± 1.23		4.85 ± 1.33	5.17 ± 1.14	4.85 ± 1.20	0.003
Identified regulation	5.41 ± 1.08	5.56 ± 1.08		5.38 ± 1.07	5.63 ± 1.03	5.37 ± 1.12	0.009
Integrated regulation	5.87 ± 0.92	5.73 ± 1.02		5.88 ± 0.95	5.95 ± 0.94	5.68 ± 0.97	<0.001
Intrinsic motivation	5.37 ± 1.15	5.55 ± 1.15	0.018	5.38 ± 1.16	5.61 ± 1.15	5.35 ± 1.14	0.004

*Values are represented as mean ± standard deviation.*

Participants who had been practicing their sport for more than 10 years (experts) showed significantly lower scores of private self-focus and lower scores of intrinsic motivation than those who had been practicing for less than 10 years (see Table 3). Conversely, participants who had practiced more during the season before lockdown reported significantly higher scores of cognitive anxiety (worry and public self-focus), perceived control, as well as external and integrated regulations of extrinsic motivation to return to sport than participants who had practiced two training sessions or less per week before lockdown (see Table 4). These results suggest that expertise (measured by the number of years of practice) seems to be associated with less self-focus when considering return to sport and that practicing more times per week seems to be associated with higher concerns while still perceiving having control on return to sport.

**TABLE 4:** Differences in anxiety and motivation between groups for weekly training sessions and training program during lockdown.

	Weekly training sessions				Training program during lockdown		
	>4 (*N* = 394)	3 or 4 (*N* = 197)	2 or less (*N* = 131)	*p*	Yes (*N* = 586)	No (*N* = 136)	*p*
Worry	3.12 ± 0.97	3.02 ± 1.01	2.83 ± 0.95	0.015	3.04 ± 0.98	3.01 ± 1.00	
Private self-focus	3.31 ± 0.69	3.24 ± 0.68	3.25 ± 0.67		3.28 ± 0.69	3.30 ± 0.67	
Public self-focus	2.89 ± 0.89	2.77 ± 0.89	2.65 ± 0.93	0.025	2.80 ± 0.89	2.88 ± 0.93	
**Total cognitive anxiety**	3.11 ± 0.64	3.01 ± 0.66	2.91 ± 0.68	0.027	3.04 ± 0.65	3.07 ± 0.70	
Somatic tension	2.23 ± 0.85	2.22 ± 0.81	2.21 ± 0.86		2.18 ± 0.81	2.40 ± 0.95	0.033
Autonomic hyperactivity	1.80 ± 0.72	1.84 ± 0.79	1.80 ± 0.71		1.77 ± 0.69	2.00 ± 0.87	0.012
**Total physiological anxiety**	2.02 ± 0.73	2.03 ± 0.74	2.01 ± 0.72		1.98 ± 0.69	2.20 ± 0.85	0.018
**Perceived control**	4.20 ± 0.64	4.06 ± 0.67	4.16 ± 0.54	0.016	4.18 ± 0.64	4.03 ± 0.58	0.001
Amotivation	1.49 ± 0.83	1.56 ± 0.88	1.45 ± 0.75		1.49 ± 0.81	1.55 ± 0.91	
External regulation	3.23 ± 1.52	3.05 ± 1.53	2.83 ± 1.47	0.018	3.10 ± 1.53	3.14 ± 1.50	
Introjected regulation	5.03 ± 1.20	4.94 ± 1.26	4.79 ± 1.17		4.97 ± 1.23	4.95 ± 1.14	
Identified regulation	5.53 ± 1.03	5.44 ± 1.16	5.31 ± 1.13		5.50 ± 1.08	5.30 ± 1.07	0.022
Integrated regulation	5.98 ± 0.87	5.68 ± 1.06	5.51 ± 0.99	< 0.001	5.85 ± 0.95	5.63 ± 1.00	0.008
Intrinsic motivation	5.46 ± 1.15	5.50 ± 1.11	5.31 ± 1.20		5.51 ± 1.13	5.17 ± 1.18	0.002

*Values are represented as mean ± standard deviation.*

Additionally, participants who had training programs during lockdown showed significantly lower scores of physiological anxiety (somatic tension and autonomic hyperactivity), significantly higher scores of perceived control over return to sport, as well as higher scores of autonomous motivational regulations to return to sport (i.e., identified and integrated regulations of extrinsic motivation and intrinsic motivation) than those who did not follow any training program (see Table 4). These results suggest that continuing sport practice at home during lockdown is associated with perceived control when considering return to sport and autonomous motivations (e.g., in line with individual’s objectives and needs) to return to sport.

Among injured participants, those who had a rehabilitation program to perform at home during lockdown (*N* = 21) showed higher scores of autonomous motivational regulations [identified (*M*_rehab_ = 5.71 ± 1.12; *M*_non–rehab_ = 5.02 ± 1.23; *W* = 130.5; *p* < 0.05) and integrated (*M*_rehab_ = 6.00 ± 0.88; *M*_non–rehab_ = 5.50 ± 0.93; *W* = 126.5; *p* < 0.05) regulations of external motivation] than those who did not (*N* = 16).

## Discussion

The aim of the present study is to investigate the differences in anxiety and motivation to return to sport across groups of competitive athletes with different levels and training habits during the COVID-19 confinement period in France. The results showed higher scores of anxiety for females, younger athletes, athletes practicing and competing at the highest level, and athletes without a training program during the lockdown. Additionally, participants who were injured at the start of the lockdown reported higher scores of cognitive anxiety to return to sport than non-injured participants.

Moreover, results suggest lower motivation scores for older athletes, experts (practicing for more than 10 years), and athletes without training programs during the lockdown. International athletes’ motivations are more controlled than national and regional athletes, and national athletes presented the best self-determined profile during the lockdown. In comparison with other levels (national and regional), international athletes show a complex motivation profile, with both high controlled and autonomous regulations. This is in line with a recent analysis of motivational processes of Olympic medalists (Jordalen et al., 2020) that recalls how much international athletes are subject to external forces. The controlling environment linked with the COVID-19 context could have catalyzed this perception of external control (e.g., financial support, athletes’ playing contract for next season).

Our results seem to be contradictory with previous studies that show lower anxiety and perceived stress among elite athletes during the COVID-19 crisis compared to either the general population or non-elite competitive athletes (Clemente-Suárez et al., 2020; Costa et al., 2020; di Fronso et al., 2020). However, in the present study, the measure of anxiety was contextualized to return to sport. The use of the TFAI-return aimed at measuring signs of anxiety regarding return to sport while athletes had no information on the future training setting and competitions’ dates. Hence, athletes competing at the highest level of competition were not sure whether they will be prepared enough for the forthcoming competition.

In previous studies, anxiety has been shown to be associated with more external regulations of extrinsic motivation to return to sport after an injury (Podlog et al., 2011). In our study, the results showed that participants displaying higher levels of anxiety also recorded higher scores of controlled motivations. This is in line with SDT (Ryan and Deci, 2017): returning to sport to avoid threats or to get external rewards is associated with anticipatory thoughts generating cognitive anxiety.

Athletes who had training programs (either from their staff or from other sources) were less anxious, perceived more control, and were more intrinsically motivated to return to sport after the confinement period (see Table 4). This is in line with theoretical models of return to sport in the context of sport injury (Wiese-Bjornstal, 2019). In fact, continuing to train, keeping in contact with the staff or other athletes, having goals and activities on a daily basis, as well as having time to train and limited extra-sport activities are ideal conditions for being confident with the capacity to return to sport with a lower loss in performance and for the pleasure of practicing sport and competing again. Furthermore, it would be of interest to record whether athletes were compliant in performing their training programs. However, it was not possible to record compliance to training programs in this study (athletes were at home on their own, so no investigators could have investigated their behaviors on a daily basis).

This study is not free of limitations. First, both psychometric questionnaires were adapted to return to sport and have not yet been validated with those adaptations. The CFA allowed the estimation of the construct validity of the TFAI-return and the SMS-return; however, external validity will need to be checked. Second, assessing coping strategies would have been of interest to estimate the usual capacity to cope with stressful events and may have been associated with lower anxiety. Likewise, the level of general stress perceived during the confinement period would have been of interest for running a more complete model of stress related to return to sport. However, we decided to target the two main variables that are known to predict return to sport (Podlog and Eklund, 2007). These variables could be the target of interventions and have been widely studied in the literature. Third, this study only investigated identified predictors of the risk of injury and impaired performance when returning to sport. Recording injuries and performances several weeks after completion of the psychometric questionnaires would have been a worthwhile addition to this study to verify if being anxious or externally motivated to return to sport after lockdown leads to increased risks of injury and impaired performance. Unfortunately, it was not possible to request the athletes to respond to a follow-up questionnaire.

The results of this study may lead to practical implications when considering the confinement period or training periods that are held at home for long periods of time (e.g., 2 weeks of self-isolation in case of COVID-19 contraction). To help athletes cope with the uncertainty of future competitions, coaches could encourage setting goals during training periods (e.g., succeeding *x* times over *y* a technical move). Goal setting has proved to be effective in reducing anxiety and increasing motivation in sport (Hogue, 2019). Additionally, anxiety reduction techniques such as breathing exercises for physiological anxiety or mental exposure using imagery for cognitive anxiety may be taught to athletes with high anxiety (van Dis et al., 2020).

Finally, we can highlight the little attention given to how coaches could be supported by federations or clubs during this lockdown. While most concerns have centered on how training programs may successfully transit from face to face to remote delivery, little attention has been given to how educators could be supported in this new unchartered territory. In this way, Orsini and Rodrigues (2020) recently gave some practical recommendations for teams working remotely for cultivating autonomous motivation in coaches as well as others in their institution, including the staff and athletes.

## Data Availability Statement

The original contributions presented in the study are included in the article/supplementary material. Further inquiries can be directed to the corresponding author/s.

## Ethics Statement

Ethical review and approval was not required for the study on human participants in accordance with the local legislation and institutional requirements. The patients/participants provided their written informed consent to participate in this study.

## Author Contributions

AR and NH conceived the study. AR, JF, MB, and NH designed the questionnaires and wrote and edited the manuscript. AR collected and analyzed the data and designed the tables. AR, MB, and NH interpreted the results of the research and the formal analysis on the successive drafts. All authors approved the manuscript in its final form.

## Conflict of Interest

The authors declare that the research was conducted in the absence of any commercial or financial relationships that could be construed as a potential conflict of interest.
